# Fault Diagnosis of Switching Power Supplies Using Dynamic Wavelet Packet Transform and Optimized SVM

**DOI:** 10.3390/s25103236

**Published:** 2025-05-21

**Authors:** Jie Xu, Jingjing Zhu, Zhifeng Wang

**Affiliations:** School of Intelligent Manufacturing and Control Engineering, Shanghai Polytechnic University, Shanghai 201209, China20221513074@stu.sspu.edu.cn (J.Z.)

**Keywords:** switching power supply, fault diagnosis, dynamic wavelet packet transform (DWPT), artificial bee colony optimized support vector machine (APABC-SVM), feature extraction

## Abstract

Switch mode power supplies (SMPSs) are prone to various faults under complex operating environments and variable load conditions. To improve the accuracy and reliability of fault diagnosis, this paper proposes an intelligent diagnosis method based on Dynamic Wavelet Packet Transform (DWPT) and Improved Artificial Bee Colony Optimized Support Vector Machine (APABC-SVM). First, an adaptive wavelet packet decomposition mechanism is used to refine the time–frequency feature extraction of the signal to improve the feature differentiation. Then, a dynamic window statistics method is introduced to construct comprehensive dynamic feature vectors to capture the transient changes in fault signals. Finally, the APABC is used to optimize the SVM classifier parameters to improve the classification performance and avoid the local optimum problem. The experimental results show that the method achieves an average accuracy of 99.091% in the complex fault diagnosis of switching power supplies, which is 21.8 percentage points higher than that of the traditional spectrum analysis method (77.273%). This study provides an efficient solution for the accurate diagnosis of complex fault modes in switching power supplies.

## 1. Introduction

As a critical component of modern electronic equipment, the stability and reliability of power supplies directly affect the operating efficiency of the entire system [1]. However, under a complex operating environment and variable load conditions, SMPSs may suffer from various failures [2], such as aging of semiconductor devices, short circuits, errors in control algorithms, and so on [2,3,4]. These failures can lead to degradation of equipment performance or even complete failure. Therefore, timely and accurate fault diagnosis and maintenance of SMPSs are of great importance to ensure the normal operation of the system.

One paper presented a new approach to nuclear power plant (NPP) fault detection and classification using wavelet transform-based artificial neural networks (ANNs). Firstly, 10 design basis accidents (DBAs) of VVER-1000 containing 15 input parameters were simulated using a multilayer perceptron (MLP) neural network and a resilient backpropagation (RBP) algorithm. Then, the time-shift properties and multi-resolution analysis properties of the wavelet transform were introduced to reduce the disturbing noise in the input training set data. The artificial neural network and wavelet transform were simulated using MATLAB R2022b software. The results show that the method improves the accuracy and speed of fault detection with high robustness [5].

To solve the problem of hard and soft fault diagnosis of active power factor correction (APFC) power supplies, another study analyzed failure modes due to the aging and failure of various sensitive components. The power supply fault waveform patterns were first analyzed based on the total harmonic distortion (THD), current ripple, and RMS of the circuit. Then, inductor current signals under various failure modes were utilized to extract and construct time–frequency fusion fault characteristics of the APFC power supply. Finally, these features were down-converted and optimized using RF algorithms. The SOA-KELM model of the APFC converter was proposed, and the feature vectors under different fault modes were utilized for fault classification and diagnosis to achieve hard and soft fault detection of the converter. The experiments showed that the method achieves 100% hard fault diagnosis accuracy and 96.36% soft fault diagnosis accuracy for the converter, which is a high diagnosis accuracy [6].

In recent years, model-based fault detection (MBFD) methods have faced diagnostic risks from multiple sources of uncertainty, such as modeling errors, measurement errors, and operational disturbances. To this end, Liao et al. constructed a risk-aware MBFD analysis framework for multilevel converters, quantitatively evaluated the probability of misdiagnosis using Monte Carlo analysis, and introduced a disturbance observer (DOB) to compensate for system parameter deviations online to achieve the robust detection of IGBT open-circuit faults. The method has strong theoretical support and practical accuracy advantages and is suitable for use in systems with clear modeling and well-developed sensors, but there are limitations in applying it to power-type systems that lack models or are difficult to calibrate [7].

The vector-constrained fault-tolerant control (FTC) method proposed in [8] is mainly applied to the operation control of single-phase cascaded H-bridge multilevel converters (CHBMCs) under open-circuit fault scenarios. The method realizes the dynamic adjustment of the voltage of healthy battery cells through online vector calculation and constraint mechanisms so as to achieve fault-tolerant operation without increasing hardware and to ensure the DC voltage balance and low harmonic output of the grid current. Its advantages are that it is insensitive to voltage level, has good scalability and real-time performance, and is especially suitable for medium- and high-voltage scenarios. However, the control strategy of this method is complicated, depends on the accurate estimation of the system state, and lacks the ability of self-adaptation and self-learning, which makes it difficult to maintain the optimal performance under the frequently changing operating conditions.

To balance the physical interpretability of model-driven methods with the strong robustness of data-driven methods, a hybrid fault detection method based on model–data fusion has been proposed. The method uses system models and historical data to jointly construct a hybrid estimator, which in turn achieves effective monitoring of sensor and actuator faults and adapts to new fault scenarios by self-updating the dataset. Experimental validation of the method on vehicle lateral acceleration sensors and motors has demonstrated its strong environmental adaptability and generalizability. However, its implementation architecture is relatively complex and places high demands on model accuracy and parameter calibration, making it difficult to deploy on low-cost devices [9].

The dynamic adaptability of feature extraction is insufficient [10,11,12]; unified methods mostly adopt fixed-parameter feature decomposition strategies, which have difficulty in effectively capturing the time-varying characteristics of fault signals under complex operating conditions [13,14]. The parameter optimization of classification algorithms is inefficient. Swarm intelligence algorithms tend to fall into local optimization, which affects the ability to discriminate multiple types of faults [15]; in addition, the reliability of multi-fault coupled diagnosis is weak [16,17,18], and the existing feature system has limited ability to characterize the scenarios of simultaneous occurrence of hard/soft faults, resulting in a high misdiagnosis rate [19].

In recent years, deep neural networks (DNNs) have been widely used in fault diagnosis due to their strong automatic feature extraction capabilities. However, such methods typically require a large number of labeled samples for training and are prone to overfitting when data are limited or expensive to obtain. In contrast, Support Vector Machines (SVMs) show stable performance in small sample scenarios, offer fast training speed, and have a simple model structure, making them suitable for use on resource-constrained devices such as embedded controllers. Therefore, this study combines Dynamic Wavelet Packet feature extraction with an Improved Artificial Bee Colony (ABC) algorithm Optimized SVM model to construct a lightweight algorithm framework suitable for switch power supply fault diagnosis. To address the above bottlenecks, we propose an intelligent diagnosis method that integrates Dynamic Wavelet Packet and Improved Swarm Support Vector Machine: (1) an adaptive wavelet packet decomposition mechanism is designed to dynamically optimize the time–frequency feature extraction process; (2) the artificial swarm algorithm is improved to enhance the parameter optimization efficiency of the Support Vector Machine.

## 2. Materials and Methods

### 2.1. Fault Set Acquisition

In this paper, a phase-shifted full-bridge (PSFB) DC-DC converter is selected as the object of study. This topology is widely used in medium- and high-power switching power supplies due to its strong control flexibility, high conversion efficiency, and high transformer utilization. The PSFB converter is highly representative due to the frequent on–off operations of the switching devices during operation, which places higher requirements on the timing identification capability and high-frequency dynamic response in fault diagnosis. Compared with the traditional buck, boost, and other simple topologies, the PSFB converter fault mode is more complex, especially when the main switching devices have short-circuit or open-circuit and other transient faults, where the impact on system operation is more significant. Although the same power device failure problem exists in other topologies, their waveform characteristics and fault propagation paths are significantly different, making it difficult to directly reuse the same set of diagnostic methods. Therefore, the selection of a PSFB converter as a research object is not only challenging but also has high research value and practical application significance.

**Figure 1:**
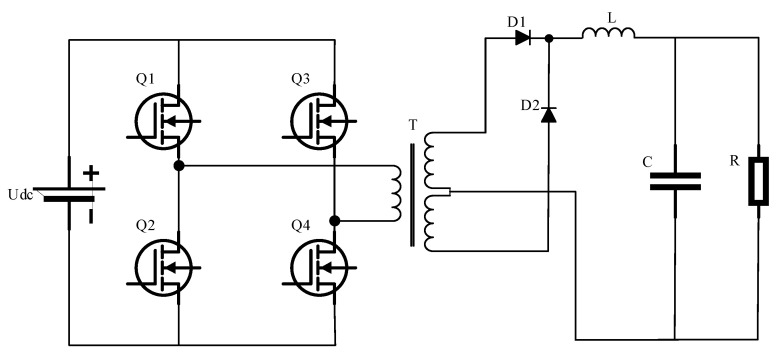
Full-bridge topology switching power supply.

This experiment uses a phase-shifted full-bridge switching power supply development board as the module to be tested; the power module primary side of the phase-shifted full-bridge structure uses low-voltage input and output methods. The full-bridge topology switching power supply core lies in the four switching tubes alternating conduction and high-frequency transformer energy transfer [20,21,22]—this is the secondary use of the center-tap full-wave rectifier, with the development of the board’s topology as shown in Figure 1.

**Figure 2:**
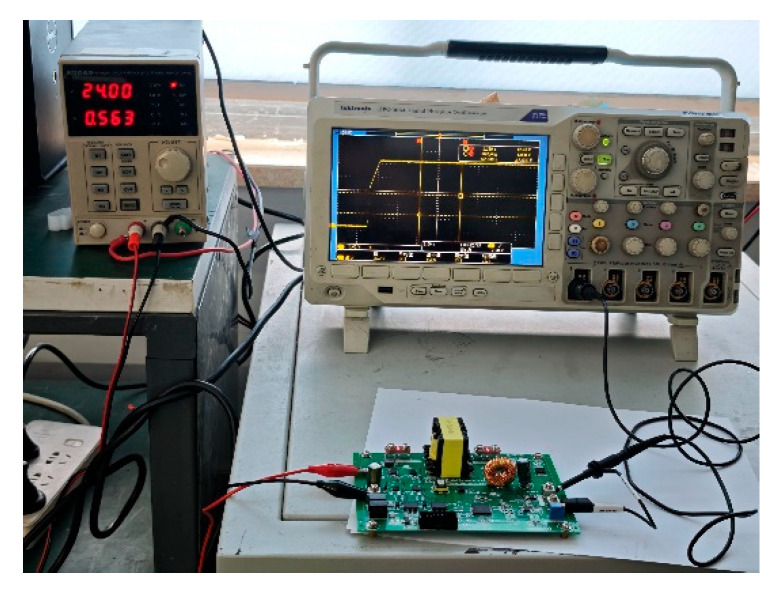
Experimental data acquisition.

A Tektronix MDO3054 oscilloscope from Tektronix, Germany was utilized in the experimental design to accurately capture the excitation response waveforms of the experimental circuits at the output end, and the waveform data were stored with the help of a USB (Lenovo, Fuzhou, China); drive. The experimental signal data acquisition environment and data acquisition flow of this section of the experiment are shown in Figure 2.

The system is in a fault-free operation in the interval where the variation in the parameter values of the circuit components is within 20%, while the system enters the fault phase when the variation is outside the 20% range. According to the failure mode shown in the figure, the test point is the change in voltage value at the output.

The present study focuses on the modeling and identification of typical failure modes. In the process of fault modeling, priority is given to the selection of key devices that have a large impact on the output waveform and high diagnostic value for fault injection. Specifically, diode D2 is in the continuity loop, its failure probability is low, and it is usually designed with a certain degree of redundancy in various control strategies. Consequently, no faults are set for it in this study. As a passive device, the performance changes of inductor L are mostly manifested as slow aging rather than sudden failures. Furthermore, its impact on the short-term response waveforms is limited. Thus, it is not included in the scope of this fault modeling.

For the primary switching components, while Q2 and Q4 are also pivotal switching elements analogous to Q1 and Q3, they form diagonally symmetric bridge arms within the phase-shifted full-bridge configuration. These components exhibit remarkably symmetrical turn-on logics, conduction pathways, and duty cycle regulation. In the empirical test, it was ascertained that the output response of Q2 and Q4 faults is highly analogous to that of Q1 and Q3 faults, and the distribution in the feature space exhibits a greater degree of overlap. In the event that Q2 and Q4 faults are configured concurrently within the sample set, there will be a substantial augmentation in the inter-class overlap. This, in turn, will result in escalated challenges during model training, the introduction of diagnostic bias, and a concomitant reduction in overall classification performance. Consequently, the short-circuit faults of Q1 and Q3 are selected as the representative fault modes in this paper. This ensures the stability and generalization ability of the model while taking into account the fault coverage and feature separability. The failure mode codes are enumerated in Table 1.

**Figure 3:**
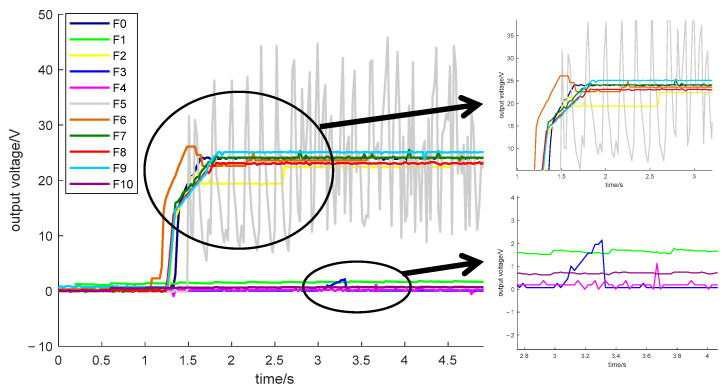
Output voltage waveform of switching power supply in each fault state.

According to the classification of the failure modes of the experimental circuit, Figure 3 shows the output waveform data obtained from the experimental circuit in the no-fault state and ten fault states. The response signal is obtained by taking the acquired waveform data with a period of 5 s.

### 2.2. Fault Feature Extraction Method

In this paper, multi-scale decomposition is performed based on adaptive Dynamic Wavelet Packet Transform, and time–frequency fusion features are constructed by combining sliding window statistics such as mean, variance, kurtosis, etc., which can effectively take into account the local time-domain variations in the signals and the global frequency-domain characteristics. The feature extraction method shows strong sensitivity to the transient disturbances and high-frequency abnormal responses commonly found in switching power supplies, and at the same time has good noise immunity and robustness, which helps to improve the accuracy of fault identification and the stability of the model. Compared with the traditional fixed-parameter wavelet transform method, the proposed DWPT has obvious advantages. Its decomposition depth and node selection have adaptive ability, and the structure is dynamically adjusted according to the actual spectrum of the signal, which can better capture the transient characteristics of the fault signal; moreover, DWPT has better time–frequency localization, which can still maintain a higher resolution when dealing with non-smooth signals and significantly improves the feature expression ability and diagnostic accuracy of the subsequent classification model.

This study makes innovative improvements in the feature extraction stage and constructs a more comprehensive fault feature vector by combining wavelet packet decomposition and dynamic feature extraction to improve the accuracy and robustness of fault diagnosis.

#### 2.2.1. Feature Extraction with Wavelet Packet Decomposition

(1) Banding for wavelet packet decomposition

A multilevel wavelet packet decomposition technique is implemented on the target voltage signal using the db4 type in Daubechies wavelet. With the help of the three-level decomposition technique, the original signal is accurately divided into eight independent frequency bands, and each node signal represents the local features of a specific frequency band, which can capture the fault features more effectively.

(2) Node energy feature extraction

The energy is calculated for each node obtained by decomposing the wavelet packet [23] according to the following formula:

(1) $$E_{i}=\sum_{n=1}^{N}{\left(wp_{i}(n)\right)}^{2}$$

where *N* is the length of the node signal, *i* is the node number, and $wp_{i}$ is the wavelet packet coefficient. These node energies reflect the strength information of the signals in different frequency bands and help to identify different failure modes.

#### 2.2.2. Dynamic Feature Extraction

To capture the time-varying characteristics and dynamic trends of the signal, the sliding window method is introduced to calculate the dynamic characteristics, including the dynamic mean and dynamic standard deviation. This method is able to reflect the variation in the statistical characteristics of the signal in a local time range, which helps to identify small error characteristics.

(1) Sliding window settings

The window size W is set to 100 and the step size S to 50 according to the signal length and sampling frequency.

(2) Dynamic Mean Calculation

The sliding window method extracts the statistical characteristics of the local signal within each window, such as mean, standard deviation, peak value, etc., by setting a fixed-length window on the time series signal and gradually sliding it. This method can effectively reflect the energy fluctuation, the degree of mutation and the dynamic characteristics of the signal in the local time period, thus realizing the real-time detection and quantification of abnormal events in non-stationary signals, which is especially effective in identifying the transient characteristics of disturbances.

The dynamic average within each window is calculated using the following formula:

(2) $$\mu_{k}=\frac{1}{W}\sum_{j=1}^{W}wp_{i}\left(j+\left(k-1\right)\cdot S\right)$$

where *k* is the window number and $wp_{i}$ is the signal at the *i*th node. The dynamic mean reflects the local trend of the signal amplitude.

(3) Dynamic standard deviation calculation

Within each window, the following equation is used to calculate the dynamic standard deviation:

(3) $$\sigma_{k}=\sqrt{\frac{1}{W}\sum_{j=1}^{W}{\left(wp_{i}\left(j+\left(k-1\right)\cdot S\right)-\mu_{k}\right)}^{2}}$$

The dynamic standard deviation reflects the degree of variation in the signal over a localized period of time and is effective in capturing anomalous changes.

(4) Skewness calculation

Skewness [24,25], as a statistical measure of deviation from the symmetry of a probability distribution, is designed to quantify the degree of asymmetry of the distribution pattern and to reveal the extent and direction of its deviation from the ideal symmetric distribution. Its mathematical definition is:

(4) $$S_{k}=\frac{E\left[{\left(X-\mu \right)}^{3}\right]}{\sigma^{3}}$$

where *X* is the random variable, mean is *μ*, the standard deviation is *σ*, and *E* is the expectation operation. The positive skewness distribution has a longer tail on the right side and the data are concentrated on the left side, indicating the presence of more high-value outliers. Negative skewness has a longer tail on the left side of the distribution and the data are concentrated on the right side, indicating the presence of more low-value outliers. Zero skewness is symmetrically distributed. By analyzing the change in skewness, the type of error and its effect on the signal distribution can be identified.

(5) Kurtosis calculation

Kurtosis is a statistic that describes the sharpness of a probability distribution or the thickness of the tails [26,27] and is a measure of the steepness of the peaks of the distribution. It is mathematically defined as

(5) $$k_{K}=\frac{E\left[{\left(X-\mu \right)}^{4}\right]}{\sigma^{4}}-3$$

where minus 3 is used to make the kurtosis of the normal distribution 0. A high kurtosis distribution with steep peaks and thicker tails indicates that there are more extreme values in the data; the low kurtosis distribution has a smooth peak and thinner tails, indicating a more even distribution of the data. Zero kurtosis corresponds to the kurtosis of a normal distribution. Kurtosis can reflect the extreme value characteristics of the signal distribution. Under normal operating conditions of the power supply, the signal kurtosis is low and the distribution is close to normal.

#### 2.2.3. Fault Feature Vector Construction

The node energy features from the wavelet packet decomposition are combined with the dynamic features to construct the final fault feature vector, F:

(6) $$F=\left[E_{0},E_{1},\ldots ,E_{i},\mu_{1},\mu_{2},\ldots ,\mu_{k},\sigma_{1},\sigma_{2},\ldots ,\sigma_{k},S_{1},S_{2},\ldots ,S_{k},k_{1},k_{2},\ldots ,k_{K}\right]$$

This feature vector, which integrates the frequency domain and time domain features, can comprehensively reflect the fault information of the signal and improve the accuracy of fault diagnosis.

The features are selected using LASSO to filter out the most useful features for classification. LASSO (Least Absolute Shrinkage and Selection Operator) is a regression method with an L1 regular term, which realizes the automatic screening of redundant or invalid features by constraining some regression coefficients to 0 in feature selection. In this paper, LASSO is used for the dimensionality reduction of extracted high-dimensional statistical feature vectors to improve the performance and stability of the diagnostic system. It not only reduces the computational complexity of the model and the risk of overfitting but also retains the key features that are most discriminative for the classification task.

**Figure 4:**
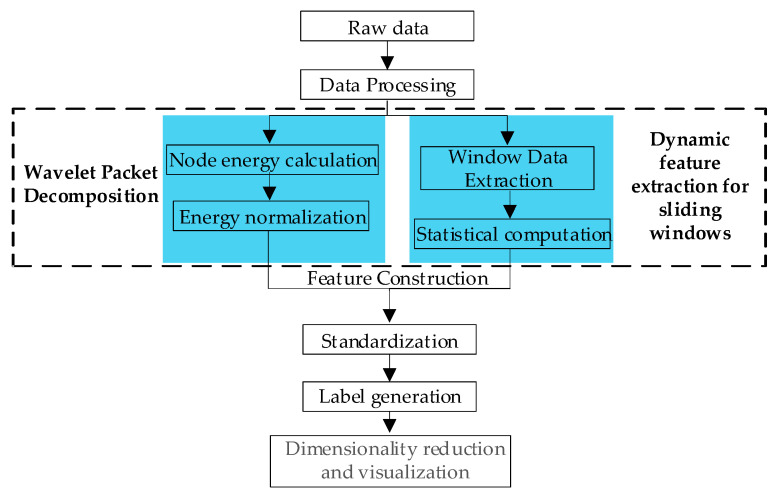
Schematic of feature extraction pipeline.

Figure 4 shows a workflow of the proposed feature extraction method. The raw data are merged with the normalized node energy of wavelet packet decomposition and the features extracted from the dynamic window, and the feature vector and visualization are constructed by t-SNE.

### 2.3. Troubleshooting Methods

**Figure 5:**
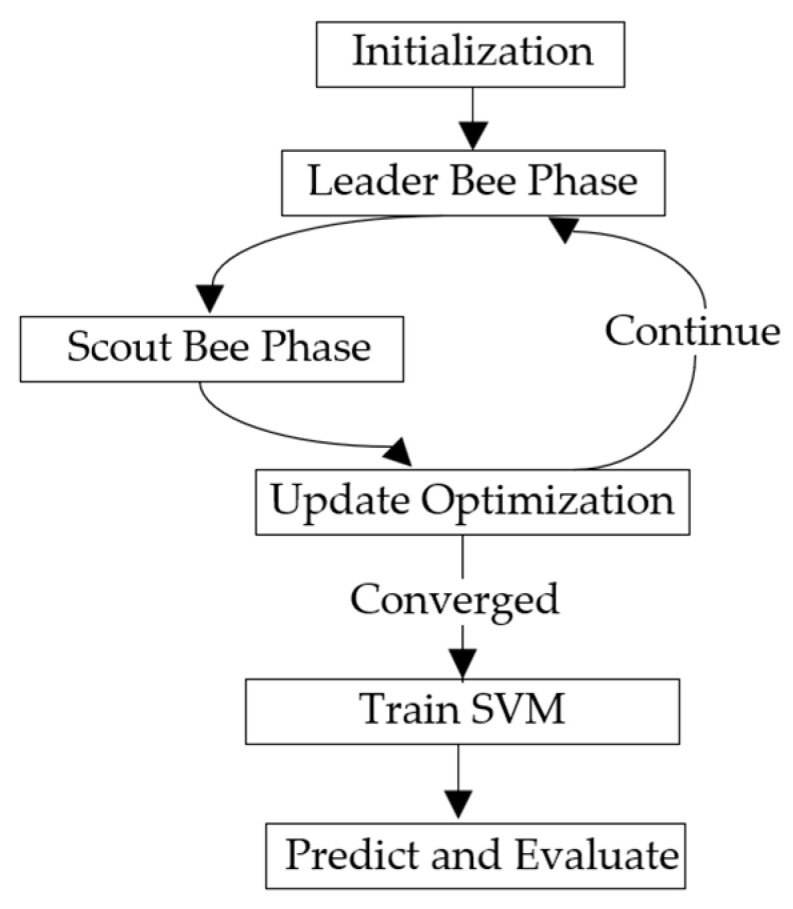
Troubleshooting method flowchart.

APABC is used to optimize the penalty coefficient C and the kernel function parameter γ of the SVM. They are used to control the error tolerance of the classification hyperplane and the distribution range of the Gaussian kernel, respectively. The classification accuracy of the SVM is used as a fitness function to find the parameter combination that gives the highest classification accuracy. The APABC algorithm flow for SVM optimization is as follows, and the flowchart is shown in Figure 5:

Step 1: initialization phase. Input the feature matrix to initialize the APABC algorithm, including setting the algorithm parameters, initializing the swarm position, and determining the dynamic adjustment factor (AP).

Step 2: guide bee phase. Guide the bees to perform a neighborhood search near the current nectar source location. A dynamic AP neighborhood search strategy is used, and part of the search uses polynomial difference learning to update the bee position. The updated nectar location is used in the following observation bee stage.

Step 3: observing bee phase. Observing bees select the honey source by a roulette based on the information provided by the leader bee. A dynamic AP neighborhood search strategy is used, and part of the search uses polynomial difference learning to further update the nectar source location.

Step 4: scout bee stage. For nectar sources that have not been updated for a long time or have low fitness, scout bees will reset their positions and randomly generate new positions to explore new potential solutions.

Step 5: updating optimization information. Evaluate whether the maximum iteration time has been reached or the system has met the convergence criteria. If the condition is met, the current optimal solution and its fitness value should be output; if the condition is not met, the iteration continues.

Step 6: training the SVM classifier. A multi-classification SVM model with RBF as the kernel is constructed using the penalty factor C optimized by the APABC algorithm and the kernel width γ of the Gaussian kernel function (RBF). The SVM training uses the SMO algorithm to automatically control the number of iterations until convergence, so there is no need to manually set the number of training rounds. The average error is calculated as a performance metric by 5-fold cross-validation after training.

Step 7: prediction and evaluation. Perform prediction on the test set and calculate the confusion matrix and evaluation metrics to evaluate the model performance.

Among them, the APABC algorithm optimizes SVM with the following features.

(1) Adaptive step-size adjustment strategy

The fixed search step size of the traditional ABC algorithm is prone to over-exploration in the late iteration, which reduces the convergence efficiency. To solve this problem, this paper proposes an adaptive step-size adjustment strategy based on exponential decreasing to balance the algorithm’s global exploration and local exploitation capabilities. By dynamically adjusting the AP factor, it allows the execution of extensive and comprehensive global search in the initial stage, followed by the transition to precise and local search in the later stage, as a way to improve the search efficiency and the quality of the optimal solution. The step factor a(t) decays dynamically with the number of iterations, defined by the formula

(7) $$AP\left(t\right)=AP_{\max}e^{\left(\frac{\ln\left(AP_{\min}/AP_{\max}\right)}{NI}\cdot t\right)}$$

where *t* is the number of iterations and NI denotes the maximum number of iterations. AP decreases exponentially with time from the initial maximum value and approaches a minimum at *t* close to NI; amax and amin are the initial and minimum step size coefficients, respectively. $T_{max}$ is the maximum number of iterations, and t is the current iteration. This design allows the algorithm to maintain a large step size early (when t is small) to improve the global search capability and to reduce the step size later (as *t* approaches $T_{max}$) to improve the local evolution accuracy.

(2) Global optimal guidance search mechanism

The traditional ABC algorithm’s following bee only relies on random neighboring individuals for the search, ignoring the historical optimal information of the population. Therefore, this paper introduces a global optimal individual guidance mechanism in the following bee stage to speed up convergence. In the following bee stage, the traditional neighborhood search is performed with 70% probability, and the search towards the current fitness optimal individuals is performed with 30% probability.

(8) $$newbee.Position=\left\{\begin{array}{l} x_{i}+\theta \cdot \left(x_{i}-x_{k}\right),\;\mathrm{if}\;\mathrm{rand}<0.7\\ x_{i}+2*rand\cdot (x_{best}-x_{i}),\;\mathrm{otherwise} \end{array}\right.$$

where $x_{best}$ is the position of the optimally adapted individual in the current population and θ is the adaptive step size coefficient. This strategy preserves the population diversity through the probabilistic hybrid search mode and also uses historical optimal information to guide the population to cluster towards the high-quality solution region.

(3) Designing exponential mapping fitness functions

A linear fitness function $F_{i}$ is used in a traditional ABC:

(9) $$F_{i}=\frac{1}{1+f\left(x_{i}\right)}$$

When dealing with high-dimensional complex problems, the range of the objective function value is too large, resulting in insufficient differentiation of the fitness. In this paper, we propose an exponential-function-based adaptation degree calculation method.

(10) $$F_{i}=\exp\left(-\frac{f(x_{i})}{MeanCost}\right)$$

where MeanCost is the current objective function mean of the population. The design is normalized to make the distribution of fitness values smoother and to avoid the dominance of extreme values on the selection probabilities.

(4) Scout Bee Local Reboot Strategy

Traditional scout bees generate new solutions completely randomly, which tends to destroy the discovered potential optimal regions. In this paper, we propose a local restart strategy based on individual history parameters to limit the search area. In the observation bee stage, the searched nectar sources are randomly crossed to learn the most nectar source locations, and the *j*th = 1,2, …, Nth dimensional component of the new nectar source vector is dynamically generated, i.e.

(11) $$x_{j}^{new}=x_{j}^{\min}+rand\left(0,1\right)\cdot \left(x_{j}^{\max}-x_{j}^{\min}\right)$$

where $x_{j}^{\max}$ represents the *j*-th dimensional component in the optimal nectar source vector and $x_{j}^{\min}$ represents the *j*-th dimensional component in the worst nectar source vector. The method uses stochastic learning of the bee colony, which searches for the worst food source on its neighboring symmetric intervals to efficiently mine the information of its neighboring nectar sources. After three cycles of updating the nectar sources, when the algorithm completes its predetermined maximum iteration period, the global optimal solution is generated as the output. The new solution is generated within the individual history search area, which preserves the local exploitation information and avoids the computational waste caused by the global blind search.

## 3. Results and Discussion

To ensure a fair comparison of different algorithms, the same circuit structure and operating parameters are uniformly used in the experimental process, including an input voltage set to 24 V, a PWM duty cycle of 50%, and a transformer ratio of 1:1. At the same time, all the fault data are sampled from the same experimental platform with a sampling rate of 10 kHz, and the length of each fault sample is 1024 points, while the feature extraction method and pre-processing process are the same. The feature extraction method is consistent with the pre-processing process. All data processing experiments are performed on the same computer, the MATLAB version is R2022a, the datasets used are 330 sample sets covering 11 typical types, and about one-third of each type is used as a test set for model performance evaluation.

### 3.1. Fault Feature Extraction

As a comparison experiment, in this section, four different feature extraction strategies are used in parallel to process the acquired excitation response signals of the test points of the experimental circuits, the signal decomposition based on WPT, the signal decomposition based on WT, and the signal decomposition based on FFT. The results of the energy analysis of the four decomposition methods are taken as the key feature indexes of the signals of the test points, and the following diagrams of the experimental circuits for different types of faults are constructed The feature vector system of the experimental circuit for each type of fault mode is constructed as shown in the figure below.

**Figure 6:**
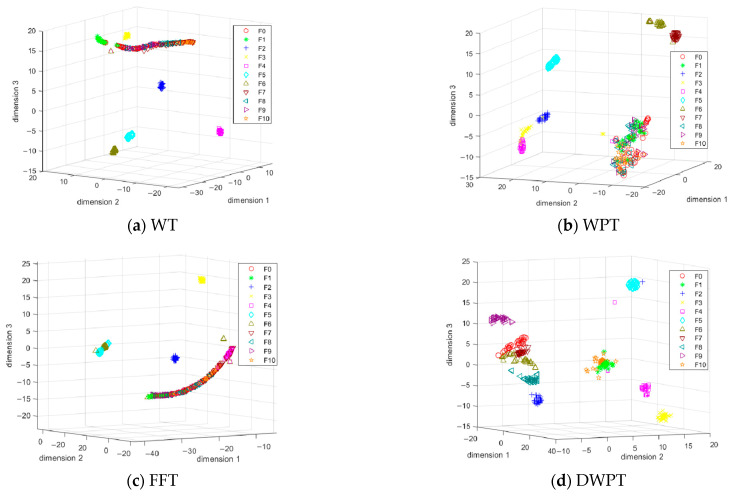
t-SNE 3D plots of the four feature extraction methods.

As shown in Figure 6, the weak separation between WT classes, the obvious overlap between DWPT fault categories, and the large intra-class dispersion indicate that the feature differentiation ability is insufficient. The global distribution of the FFT is loose, some of the fault categories are completely mixed, and independent clusters cannot be formed. After the features extracted based on DWPT are downscaled by t-SNE, different fault categories show significant separation in 3D space, and the contour coefficients [28,29] of DWPT are 0.83 and 1.28 with the average intra-class distance, and those of WPT are 0.75 and 1.34. The contour coefficients of DWPT are improved by about 10.67% compared with those of WPT, indicating that its clustering effect is better, and similar samples are tighter and clearer inter-class boundaries. The intra-class distance of DWPT decreases by 4.48%, indicating that the clustering of similar faulty samples is significantly improved. This verifies the improved extraction capability of DWPT for fault-sensitive features from a visualization perspective, and the feature extraction capability of these four methods will be verified with classifiers later.

### 3.2. Troubleshooting Experiment Results

The samples obtained from the experiments are divided into one-third as the test set and two-thirds as the training set. Four fault diagnosis methods with different classifiers, ABC-SVM, APABC-SVM, SVM, PSO-SVM, and Decision Tree combined with DWPT feature extraction, are compared to evaluate their performance differences in fault diagnosis. The swarm size npop of the APABC algorithm and ABC algorithm is set to 20, and the maximum number of iterations NI is 100 for both. The particle swarm size of the PSO algorithm is set to 20, and the maximum number of iterations is 100. The g and γ of the SVM are set to a constant 1 and 0.5, respectively. The correct rates are obtained as shown in Table 2.

**Figure 7:**
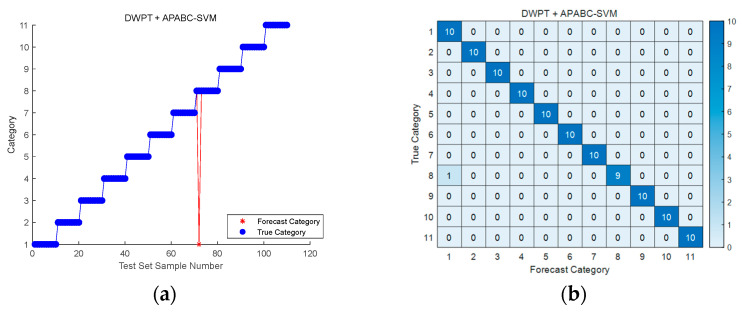
Plot of test sample results for the methodology of this paper: (**a**) Comparison of DWPT + APABC-SVM classification prediction results; (**b**) DWPT + APABC-SVM Fault Classification Confusion Matrix.

Figure 7a shows the test set classification results of the DWPT + APABC-SVM method for 11 classes of defect patterns (F0–F10). The horizontal axis is the number of test samples, and the vertical axis is the fault category label. The results show that the overall classification accuracy is as high as 99.091%, which verifies the reliability of the method in multi-class fault diagnosis. The separability between fault classes is significant, and the sample points of key faults, such as capacitor aging, MOS tube breakdown, etc., are all tightly clustered in the corresponding class area without cross-class overlap; the sample number F10 simultaneous faults are still accurately classified thanks to the decoupling ability of DWPT for time–frequency coupling features. The diagonal elements of the confusion matrix in Figure 7b account for >98%, indicating that the majority of the predicted categories are consistent with the real categories; the main misclassification is focused on the resistance change, which results from the partial overlap of the frequency bands of the two fault features.

In a comparison of the four different feature extraction methods, DWPT outperforms all classification methods. When combined with APABC-SVM, the accuracy rate is 99.091%, which is significantly better than other feature extraction methods. Even when using the basic classification method, the accuracy rate is still higher than the traditional methods. This is because DWPT is able to adaptively capture the transient features of the signal by dynamically adjusting the number of decomposition levels, which improves the ability to characterize complex fault modes. WPT has the worst performance. The fixed decomposition structure of WPT leads to a weak correlation between features and fault modes, resulting in feature redundancy or information loss, which weakens the classifier’s discriminative ability. It makes APABC-SVM overfitting noise on low-quality feature sets. FFT only provides global frequency domain information and cannot localize the transient features of time-varying faults, so the correct rate is lower. WT performs moderately well. Wavelet transform has time–frequency localization, but the basis function is fixed, which is insufficiently sensitive to high-frequency transient features.

In addition to the accuracy rate, this paper further analyzes the training time and testing time as complementary evaluation metrics, as shown in Table 2. The DWPT + APABC SVM method proposed in this paper achieves excellent overall performance. Although APABC is slightly more computationally intensive, it maintains low training and testing times and achieves the highest classification accuracy, balancing diagnostic accuracy with computational efficiency. In contrast, the traditional SVM performs poorly in terms of diagnostic ability with an accuracy of 72.73%, while the Decision Tree, although fast in testing, has a significantly lower accuracy of 86.364%, showing signs of overfitting. Other classifiers, such as PSO-SVM or Unoptimized SVM, although faster to train, did not have good accuracy.

For different classifiers, APABC-SVM achieves the highest correct rate in DWPT, and the optimization algorithm improves the accuracy by 26.36% compared to the base SVM. The correct rate of ABC-SVM is slightly inferior to that of APABC-SVM, although its performance is excellent, indicating that the improved strategy further enhances the efficiency of parameter optimization. The correct rate of PSO-SVM is 91.818%, indicating that the particle swarm algorithm has certain limitations, easily falls into local optimization, and has weak adaptability to complex feature distribution. The correct rate of the basic SVM is only 72.73%, and its kernel function parameters are fixed, which makes it difficult to adapt to the multi-scale characteristics of dynamic features.

Furthermore, under the same classification framework, different feature extraction methods are compared. The results show that DWPT is the key to improving diagnostic accuracy by providing highly discriminative local time–frequency features. The combination of DWPT and APABC-SVM forms an effective feature–classifier synergy. Traditional methods like FFT + APABC-SVM only reach 77.273% accuracy; even with advanced classifiers, poor feature quality remains a bottleneck.

The experimental results show that the method in this paper demonstrates balanced performance, adaptability, and diagnostic accuracy under the given dataset, verifying its potential for application in the intelligent diagnosis of small samples of power supply faults. The synergistic optimization of feature extraction methods and classifiers is the core of improving fault diagnosis accuracy.

## 4. Conclusions

This study draws the following innovative conclusions by systematically comparing the combined performance of various feature extraction and classification methods.

At the feature extraction level, DWPT effectively solves the frequency band aliasing problem of traditional static decomposition methods in non-smooth signal processing by introducing an adaptive decomposition mechanism. Experiments show that the method can significantly improve the feature differentiation, improve the contour coefficient by 10.67%, reduce the intra-class distance by 4.48%, and successfully separate the complex failure modes, such as the saturation of the magnetic element and the open circuit of the rectifier bridge, through the time–frequency analysis of the voltage dip signal in the fault diagnosis of switching power supply components. At the classifier optimization level, the proposed APABC-SVM algorithm improves the neighborhood search strategy and dynamically adjusts the selection probability so that the SVM achieves an accuracy of 99.091% in the multi-class fault classification of the switching power supply, which is 26.361% higher than the standard SVM.

DWPT significantly improves the feature quality through adaptive decomposition, which is the key to improving the diagnostic accuracy, and APABC-SVM improves the search strategy to bring the classification performance of SVM close to the theoretical limit. The combination of DWPT and APABC-SVM realizes the closed-loop optimization of “dynamic feature-intelligent classification” and provides a new paradigm for complex fault diagnosis.

Due to experimental limitations, the test set contains a relatively small number of samples per fault type. However, since all algorithms are evaluated under identical conditions, the comparison remains fair. The results confirm that the proposed DWPT + APABC-SVM method maintains high accuracy even with limited data, proving its effectiveness.

Future work will focus on the fusion of multi-physical field signals, such as heat and magnetism, with DWPT to further enrich the feature dimensions and develop lightweight APABC-SVM algorithms to meet the real-time requirements of edge computing devices.

## Figures and Tables

**Table 1:** Failure mode codes.

Trouble Code	Defective Element	Failure Mode
F0	-	trouble free
F1	MOSFET Q1	short circuit
F2	MOSFET Q1	open circuit
F3	Diode D1	short circuit
F4	Diode D1	open circuit
F5	Capacitor C	open circuit
F6	Capacitor C	reduced capacity
F7	Capacitor C	increased capacity
F8	Resistor R	reduced resistance
F9	Resistor R	increased resistance
F10	MOSFET Q1Q3	short circuit

**Table 2:** Result of fault diagnosis by methods.

Feature Extraction Methods	Classification Method	Training Time/s	Test Time/s	Accuracy
DWPT	Decision Tree	0.5402	0.0503	86.364%
DWPT	SVM	0.2909	0.1070	72.73%
DWPT	PSO-SVM	0.2376	0.1117	91.818%
DWPT	ABC-SVM	0.3072	0.1108	98.182%
DWPT	APABC-SVM	0.2309	0.1018	99.091%
FFT	APABC-SVM	0.1675	0.0135	77.273%
WPT	APABC-SVM	0.1714	0.0152	59.091%
WT	APABC-SVM	0.1789	0.0153	79.091%

## Data Availability

The data presented in this study are available upon request from the corresponding author. The data are not publicly available due to privacy restrictions.

## Abbreviations

The following abbreviations are used in this manuscript:

SMPS	Switching Mode Power Supply
SVM	Support Vector Machine
DWPT	Dynamic Wavelet Packet Transform
FFT	Fast Fourier Transform
WPT	Wavelet Packet Transform
WT	Wavelet Transform
PSO-SVM	Particle Swarm Optimized Support Vector Machine
APABC-SVM	Adaptive Improved Artificial Bee Colony Optimized Support Vector Machine
ABC-SVM	Artificial Bee Colony Optimized Support Vector Machine
